# Versatile multiple object tracking in sparse 2D/3D videos via deformable image registration

**DOI:** 10.1371/journal.pcbi.1012075

**Published:** 2024-05-20

**Authors:** James Ryu, Amin Nejatbakhsh, Mahdi Torkashvand, Sahana Gangadharan, Maedeh Seyedolmohadesin, Jinmahn Kim, Liam Paninski, Vivek Venkatachalam

**Affiliations:** 1 Department of Physics, Northeastern University, Boston, Massachusetts, United States of America; 2 Department of Neuroscience, Columbia University, New York, New York, United States of America; Brown University, UNITED STATES

## Abstract

Tracking body parts in behaving animals, extracting fluorescence signals from cells embedded in deforming tissue, and analyzing cell migration patterns during development all require tracking objects with partially correlated motion. As dataset sizes increase, manual tracking of objects becomes prohibitively inefficient and slow, necessitating automated and semi-automated computational tools. Unfortunately, existing methods for multiple object tracking (MOT) are either developed for specific datasets and hence do not generalize well to other datasets, or require large amounts of training data that are not readily available. This is further exacerbated when tracking fluorescent sources in moving and deforming tissues, where the lack of unique features and sparsely populated images create a challenging environment, especially for modern deep learning techniques. By leveraging technology recently developed for spatial transformer networks, we propose ZephIR, an image registration framework for semi-supervised MOT in 2D and 3D videos. ZephIR can generalize to a wide range of biological systems by incorporating adjustable parameters that encode spatial (sparsity, texture, rigidity) and temporal priors of a given data class. We demonstrate the accuracy and versatility of our approach in a variety of applications, including tracking the body parts of a behaving mouse and neurons in the brain of a freely moving *C. elegans*. We provide an open-source package along with a web-based graphical user interface that allows users to provide small numbers of annotations to interactively improve tracking results.

## Author summary

While deep learning with convolutional neural networks has been successfully applied to many multiple object tracking problems, these advances do not immediately generalize to videos of fluorescence reported dynamics in living tissue, where the combination of sparse global distributions and locally dense, homogeneous peaks present a challenging instance of a multiple object tracking problem. Imaging such sparse fluorescent signals is a standard tool for observing neuronal activity in genetically engineered animals, and performing imaging in naturally behaving animals to place that activity in the context of behavior only increases the difficulty of the problem. Thus, this step is typically a significant bottleneck in efforts to understand the relationship between neuronal activity and naturalistic behavior.

We build upon recent advances in spatial transformers and differentiable grid sampling to develop a new registration-based approach: ZephIR, a semi-supervised multiple object tracking algorithm with a novel cost function that can incorporate a diverse set of spatio-temporal constraints that can change dynamically during optimization. Local registration of image features enables tracking of keypoints even in sparse imaging conditions, such as fluorescent cellular data, while a spring network incorporates a flexible motion model of the neighboring keypoints without the need for a highly specialized skeletal model. Feature detection can help fine-tune tracking results to match a nearby detected feature in the image or even recover good tracking accuracy in cases where registration clearly fails to produce good gradients. In addition, any amount of new manual labor, whether simply verifying correct results or fixing incorrect ones, can dramatically improve ZephIR’s accuracy. Through this workflow, ZephIR achieves state-of-the-art accuracy with minimal manual labor, even on a freely behaving *C. elegans*, where large deformations present a particularly challenging tracking problem.

This is a *PLOS Computational Biology* Software paper.

## Introduction

Imaging sparse fluorescent signals has become a standard tool for observing neuronal activity. To place that activity in the context of behavior, it becomes increasingly important to perform that imaging in naturally behaving animals [1]. Tracking the fluorescent sources through the moving and deforming tissue of these behaving animals is a challenging instance of a multiple object tracking (MOT) problem, and this step is typically a bottleneck for extracting clean measures of activity [2].

Recently, deep learning with convolutional neural networks has been leveraged for many MOT problems with video data including controlling self-driving cars, inferring postural dynamics in humans and animals (DeeperCut [3], DeepLabCut [4], etc. [5]), and computational video editing (non-tracking CGI problems). These advances don’t immediately generalize to videos of fluorescence reported dynamics in living tissue for several reasons.

(1) In contrast to applications like human or vehicle tracking where each object has unique identifiers that can be exploited, two fluorescence signals in the same video are often generated by nearly identical sources and therefore lack distinguishable features [4–7]. (2) While transfer learning has been successfully implemented in scientific applications involving natural videos (a horse galloping) [4, 8], the low-level spatial and temporal features detected by these networks rarely reflect structures found in fluorescence microscopy data [9, 10]. Thus, this approach rarely reduces the quantity of additional training data required for such applications [4, 11]. Approaches that successfully reduce training data must make hard assumptions about the underlying structure via direct parameter reduction, regularization, or data augmentation [12–15]. (3) Typical applications of convolutional neural networks analyze images composed of many discriminable textures that fill the image space [16]. Fluorescence microscopy data, however, often has regions of interest with similar fluorescent cells surrounded by voids of black pixels. The combination of sparse global distributions and locally dense homogeneous peaks are less well-suited to convolutional networks, as it becomes harder for convolutional networks to extract useful features for downstream tasks [17, 18]. Some methods are proposed to improve the performance of convolutional networks on sparse data but their utility is not shown in the context of MOT [17, 19].

Some of the issues mentioned above may be alleviated by exploiting existing temporal structures in most biological systems. With sufficiently immobilized animals or high recording frame rates, temporal information can be used to search the vicinity of a cell’s previous location and match identities by minimizing displacement over time. However, motion often provides critical context for the problem being investigated (e.g. imaging neuronal dynamics to understand behavior [20]). On the other hand, capturing such motion can often preclude achieving a high frame rate, especially when serially imaging slices of a volume or attempting to recover a signal from a dim fluorescent source. In these cases, it becomes beneficial to restrict a motion model with relevant biological constraints, such as maintaining relative positions of cells.

### Relevant work

Cell tracking methods can be categorized into the following two groups: (1) detect and link, and (2) registration-based. Detect and link algorithms have two distinct steps [6, 11, 12, 14, 21, 22]: (a) Detection, where identity-blind candidate locations for objects are proposed by a segmentation or keypoint detection algorithm at each time frame independently. (b) Linking, where temporal associations between detected objects are determined to establish a single continuous worldline across all frames for each individual object. A major drawback of this two-step approach is the propagation of errors from the detection step. Errors that occur in the detection step are difficult to recover from, and they can have detrimental effects on linking and overall tracking quality. Several linking methods have been proposed that are robust to detection outliers, but they either require training with large amounts of manually or synthetically produced ground-truth data, or are not scalable to lengthy videos [11, 21].

An alternative approach is registration, which directly operates in the image space and optimize some transformation parameters that align a frame to some other frame [4, 15, 23–27]. This is done by mapping the underlying image grid from the source to the reference space using the transformation parameters and interpolated pixel values. The transformation parameters must be optimized for each new image over a number of iterations.

Fortunately, recent advances in spatial transformers and differentiable grid sampling have dramatically decreased computational burden and increased performance via GPU acceleration for registration-based approaches [24, 27–30]. Similarly, modern optimization packages such as PyTorch allow the construction of dynamic computational graphs that support more complex nonlinear transformation families and novel cost functions with various regularizers.

Here, we build upon these recent advances to develop a new registration-based approach: ZephIR, a semi-supervised multiple object tracking algorithm with a novel cost function that can incorporate a diverse set of spatio-temporal constraints that can change dynamically during optimization. Our proposed method is capable of efficiently and accurately tracking keypoints in a wide range of 2D or 3D videos. It allows the user to tune a number of parameters controlling the relative strengths of the registration loss and other constraints, and hence generalizes well to a wide range of biological assumptions. To showcase the efficacy and versatility of our method, we demonstrate its performance on a number of biological applications, including cell tracking and posture tracking.

## Design and implementation

### Frame sorting

ZephIR tracks a fixed set of keypoints within a volume over time by matching keypoints between an annotated *reference* frame and an unlabeled *child* frame. To fully analyze a movie, we need to register every frame to a reference frame.

**Fig 1:**
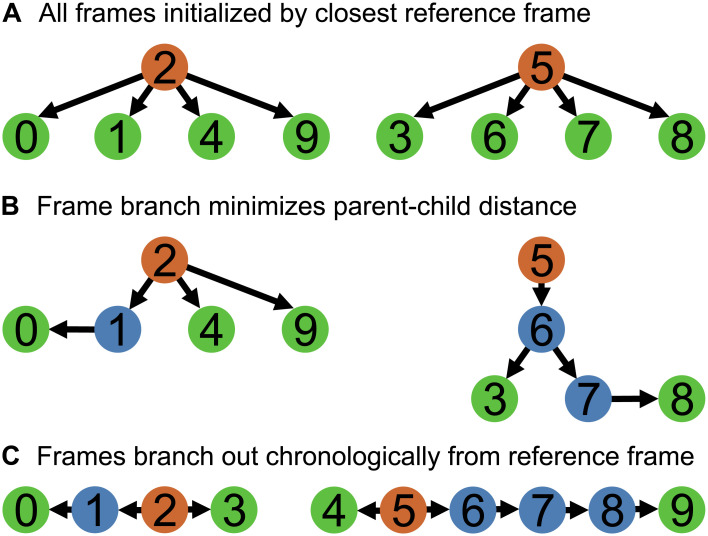
Overview of frame sorting strategies. Orange indicates fully annotated reference frames, blue indicates parent frames with at least one child frame, and green indicates child frames. **A:** In the simplest strategy, all frames are initialized by the closest reference frame. **B:** Frames are sorted into ordered queues based on similarity. Each of these branches start with a reference frame, and new child frames are added such that the parent-child similarity distance is minimized, naturally clustering similar frames around each reference frame. **C:** Frames are sorted chronologically, branching both forward and backwards from each reference frame.

For many datasets, it is best to register every child frame directly to a coarsely similar reference frame, and let annotations for that reference frame provide initial guesses for keypoints in the child frame (Fig 1A). For this, we must identify a set of representative reference frames that capture the range of deformation patterns present in the movie, and we must assign each remaining frame to one of those reference frames. A pairwise distance between all pairs of frames is determined by a similarity metric (e.g. correlation coefficient) applied to low-resolution thumbnails. A k-medoids clustering algorithm is applied in the chosen “similarity” space to identify a small number of median frames to best serve as reference frames for all other frames in the corresponding cluster (Fig 1A and S2 Fig) [4, 25].

In other datasets, the tracking results from one frame may provide useful insight into the solution for another frame. For example, a frame that is close (in similarity space) to the reference may be easy to track. The tracked results from that frame, in turn, may provide a better initial guess for keypoints in a frame that is further away from the reference. This can reduce the distance between the initial guess and correct positions as well as the difficulty of the optimization problem (S2 Fig). Thus, every child frame being registered is associated not only with a reference frame (a registration target), but also a previously registered *parent frame*, which provides the initialization prior to optimization (Fig 1B).

Additionally, the learning rate offers a way to improve the optimization trajectory by controlling the rate at which the optimized parameters, $\theta_{i}^{\left(c\right)}$, are updated after each registration iteration. The learning rate for the child frame is partly determined by the distance between the parent and child frames. We expect that when a parent-child pair are close in the similarity space, the keypoints do not undergo significant displacements. Hence, given a set number of registration iterations, we may apply a lower learning rate for a similar parent-child pair to limit displacements between the two frames, and scale up to a higher learning rate in the case of a dissimilar pair to allow tracking of features much further away from the initial coordinates. The combination of these effects produces a flexible limit on the range of possible optimization results for the child frame based on coarse similarity to its parent frame [31–33].

To take full advantage of this parent-child interaction, we sort all frames into distinct sequences of parent-child frames based on similarity. Each of the resulting *branches* begin from a previously selected median reference frame. The subsequent child frames are selected to minimize the distance from a parent frame until every frame is assigned to a branch. Doing so produces unique sets of frames that stem from each reference frame, naturally forming clusters that separate similar frames from dissimilar ones. This is particularly useful for datasets that repeatedly sample from a limited set of postures or global spatial structures (e.g. locomotion).

However, not all datasets have temporal patterns that can reliably make use of the similarity-based initialization method. For such datasets, a chronologically sorted queue may be more reasonable and provide better accuracy overall, where a branch simply stems from each reference frame both forwards and backwards in time until it encounters the first frame, the last frame, or another branch (Fig 1C). Note that the parent-child interactions during tracking are still the same regardless of the sorting method. For a chronologically sorted queue, the controlled variation of learning rates effectively allows us to adapt to different capture frame rates. A high frame rate video often captures smooth motion that benefits from low learning rates but a low frame rate video does not.

ZephIR tracks all keypoints in an image or volume (Fig 1A) simultaneously before moving onto a subsequent frame in the sorted queue (Fig 1B). This matching is done by minimizing a loss function L with four contributions:

$$L=\lambda_{R}L_{R}+\lambda_{N}L_{N}+\lambda_{D}L_{D}+\lambda_{T}L_{T}=\lambda \cdot L$$

We measure overlap of local image features around the keypoint via $L_{R}$. We measure relative elastic motion between keypoints via $L_{N}$. We measure the distance of each keypoint to the nearest candidate location from a precomputed set via $L_{D}$. We measure smoothness of keypoint-determined dynamical features (e.g. fluorescence or motion) via $L_{T}$. Each is described in more detail below.

The relative weights of each term, **λ**, can be freely adjusted by the user to better fit a particular dataset. The user can also set the relative weights to change while tracking a single frame to allow the algorithm to shift focus to different loss components over a number of optimization iterations.

### Image registration, $L_{R}$

The first term of our algorithm measures overlap of local image descriptors.

**Fig 2:**
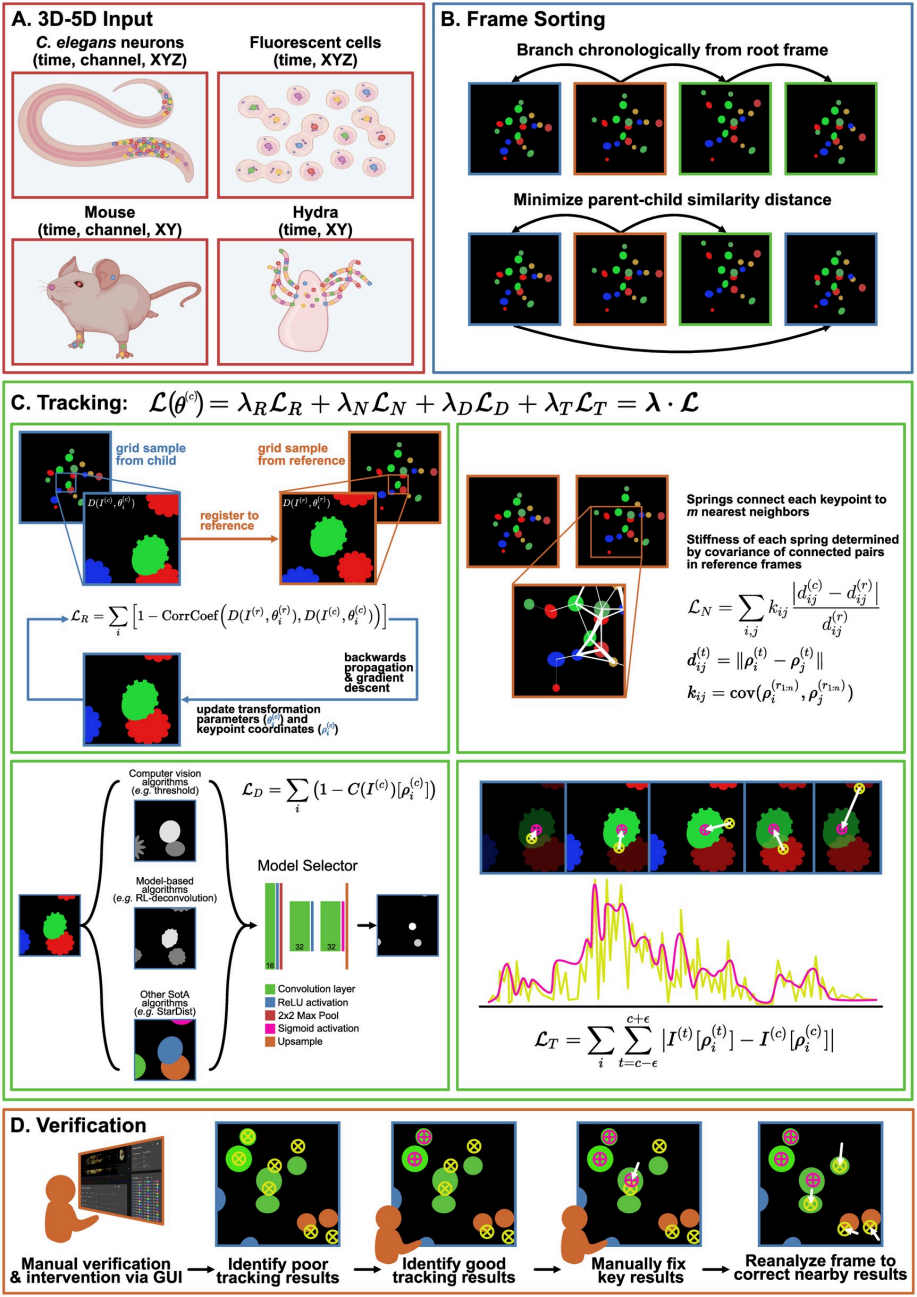
Overview of ZephIR algorithm. **A:** Examples of input datasets, created with BioRender.com. ZephIR can track keypoints in various biological systems, including fluorescent cellular nuclei in a tissue and body parts that summarize a posture. Input dimensions can range from 3D (time, XY) to 5D (time, channel, XYZ). Colored dots indicate example keypoints to be tracked. **B:** Frame sorting schemes. A branch defines an ordered queue of frames to be tracked. Each branch begins at a manually annotated reference frame (orange), but subsequent parent (blue) and child (green) frames in a single branch can be sorted either by chronology (top) or by minimizing the similarity distance between each parent-child pair (bottom). **C:** Overview of tracking loss. Tracking loss is comprised of four terms: 1) $L_{R}$ (top left), overlap of local image features around each keypoint, sampled from the current frame and its nearest reference frame, 2) $L_{N}$ (top right), elastic connections between neighboring keypoints with varying stiffnesses based on covariance of the connected keypoints, 3) $L_{D}$ (bottom left), proximity to features detected by a shallow model selector network that takes in a number of existing feature detection software as input channels, 4) $L_{T}$ (bottom right), smoothness of temporal dynamics at each keypoint position. **D:** Overview of steps for manual verification and additional supervision. Users can verify tracking results as correct or identify incorrect results. After fixing a few key incorrect results, ZephIR can use those new annotations as well as the verified correct tracking results to improve tracking results for all other keypoints in that frame (and all its child frames).

For each keypoint *i* in a child frame, *I*^(*c*)^, we generate a sampling grid centered around that keypoint’s coordinates in 3D space, $\rho_{i}^{\left(c\right)}$ (origin at frame center, with fixed *z* = 0 for 2D images). A sampling grid defines a map for a pixel (or an interpolated subpixel) from the original image to each pixel of an output image of the same spatial shape as the sampling grid, therefore producing arbitrary transformations of the image, such as simple crops or complex nonlinear deformations. We define a set of transformation parameters, $\theta_{i}^{\left(c\right)}$, that is closely related to $\rho_{i}^{\left(c\right)}$ but may include additional transformation models, such as rotation, to characterize and construct a sampling grid centered at the keypoint coordinate. We use the grid to sample from the child frame accordingly and produce a representation of the local image information, i.e. an image descriptor, around each keypoint (Fig 2C, top left, blue): $D(I^{\left(c\right)},\theta_{i}^{\left(c\right)})$.

The sampled descriptors are foveated to prioritize more local information relative to the neighboring features. In lieu of image pyramids [34], we apply a Gaussian blur to reduce the effective resolution of the descriptors at the start of optimization. The resolution is dynamically increased every few registration iterations as the magnitude of the blur is decreased. We empirically observed that doing so avoids gradient values that are too small or large, both of which can occur in regions with sharp, well-defined edges surrounded by a uniform background. On the other hand, restoring the original resolution of the image still provides the best available information for fine-tuning tracking results towards the end of the optimization loop.

Similarly, a set of reference descriptors that serve as registration targets are sampled from a reference frame, *I*^(*r*)^. The user-defined annotations for that reference frame are used to calculate fixed set of transformation parameters, $\theta_{i}^{\left(r\right)}$, and thus a fixed set of sampling grids that generate a reference descriptor centered around each annotation (Fig 2C, top left, orange): $D(I^{\left(r\right)},\theta_{i}^{\left(r\right)})$.

Using the two sets of image descriptors, our registration loop optimizes the transformation parameters for the child frame, $\theta_{i}^{\left(c\right)}$, to minimize the following loss term:

$$\begin{array}{c} L_{R}\left(\theta^{\left(c\right)}\right)=\sum_{i}[1-\text{CorrCoef}(D\left(I^{\left(r\right)},\theta_{i}^{\left(r\right)}\right),D\left(I^{\left(c\right)},\theta_{i}^{\left(c\right)}\right))] \end{array}$$

The optimized parameters $\theta_{i}^{\left(c\right)}$ are then used to calculate the desired results, the keypoint coordinates for the child frame, $\rho_{i}^{\left(c\right)}$. Note that these coordinates are also used for different loss components below, but as $\rho_{i}^{\left(c\right)}$ is calculated from $\theta_{i}^{\left(c\right)}$, gradients are always accumulated at $\theta_{i}^{\left(c\right)}$.

### Spatial regularization, $L_{N}$

Cellular motion within a tissue tends to be highly correlated, but these correlations can be hidden in sparse fluorescent movies that only highlight a small number of cells (or subcellular features) [35]. Even in less sparse movies, correlations between nearby keypoints may not be well-captured by descriptors, especially when deformations, noise, or lighting conditions prevent descriptor alignment. In order to maintain a similar spatial structure during motion without relying on highly specialized skeletal models, such as those used in animal posture trackers [36–38], we add an elastic spring network between neighboring keypoints [35, 39, 40]. The resulting penalty to relative displacement of neighboring keypoints prevents unreasonable deformations, providing a simple and flexible spatial heuristic of the global structure and motion present in the data (Fig 2C, top right).

Despite ZephIR tracking a fixed number of keypoints across the video, the spring network makes it robust to fluctuations in the number of keypoints visible in a frame. In frames where a keypoint may not be visible or present, it fails to produce useful image descriptors for registration, but the spring connections to its neighboring keypoints allow us to keep track of its approximate location.

Each of the *i* keypoints being tracked is connected to *j* nearest neighbors to define the following loss term:

$$L_{N}=\sum_{i,j}k_{ij}\frac{\left|d_{ij}^{\left(c\right)}-d_{ij}^{\left(r\right)}\right|}{d_{ij}^{\left(r\right)}}$$

where

$$d_{ij}^{\left(t\right)}=\left\|\rho_{i}^{\left(t\right)}-\rho_{j}^{\left(t\right)}\right\|$$

describes the distance between keypoints *i* and *j* in the frame *t*.

The stiffness of each spring connection, *k*_*ij*_, is initially set to a constant 1.0 for all connections. However, when multiple reference frames are available, *k*_*ij*_ is further adjusted to better model the spatial patterns in the data by accounting for the covariance of connected keypoints across all available *n* reference frames, *r*_1:*n*_:

$$k_{ij}=\text{cov}\left(\rho_{i}^{\left(r_{1:n}\right)},\rho_{j}^{\left(r_{1:n}\right)}\right)$$

This ensures that connections between highly covariant keypoints are made stronger while connections between keypoints with more weakly correlated motion are weakened or cut accordingly.

### Feature detection, $L_{D}$

For this component of the algorithm, we solve an easier problem of identity-blind feature detection, as such detection algorithms have been shown to be fruitful in the context of tracking [6]. Namely, we identify key features (such as the center of a cell) present in a volume *without* matching them to a specific feature in some other volume. Adding this information allows us to dynamically combine both registration and detect-and-link strategies into a single loss function.

This object or feature detection problem has been well-studied, and a wide variety solutions have been proposed. Solutions can range from more parameter-free algorithms (e.g. Richardson-Lucy deconvolution [41, 42]), to algorithms requiring more fine-tuning (e.g. Viola-Jones [43]). More recently, deep convolutional neural networks have shown to be powerful, effective solutions as well (e.g. StarDist [10]). Importantly, each of these approaches may work better or worse on different classes of images. Generalization to new datasets can be hard to predict, especially for neural networks that are trained on data generated from a single source.

Our approach is to automatically evaluate simple combinations of these established algorithms by using a simple model-selecting network. After identifying a set of candidate models, we provide the outputs of these models as input channels to a small convolutional neural network (CNN), capable of producing optimized combinations of some or all of the selected models. If a particular model is best suited for a dataset, network weights for the corresponding input channel are increased during training while suppressing other channels, thus it is not critical for the user to select an “optimal” set of models since even one well-suited model can sufficiently increase performance. The model selector network can be trained using any fully annotated frame, and the low number of learnable parameters in the network also allows fast training for each new type of data or imaging condition, which in turn allows rapid experimentation with new selections of models to test as inputs.

The ultimate output of this selector network, *C*(*I*^(*c*)^), is formulated as a probability map, where each pixel of the original image is assigned some probability of being a desired feature (Fig 2C, bottom left). By indexing the map to obtain the assigned probability at the keypoint coordinates, $\rho_{i}^{\left(c\right)}$, we use this information to push tracking results towards detected features:

$$L_{D}=\sum_{i}(1-C\left(I^{\left(c\right)}\right)\left[\rho_{i}^{\left(c\right)}\right])$$

### Temporal smoothing, $L_{T}$

Given a sufficiently fast imaging rate, we expect pixel intensity values to be smooth across a small local patch of frames, even for cellular datasets where pixel intensities represent smoothly-varying dynamical signals [44, 45]. Thus, we attempt to maintain smoothly-varying local pixel intensities as a form of temporal regularization. For datasets where expected dynamics are appreciably slower than the imaging rate, the strongest version of this regularization is to penalize any deviation from a local zeroth-order fit. We apply this across a small (*ϵ* ≈ 2 frames) patch of frames from *c* − *ϵ* to *c* + *ϵ* that are registered simultaneously, and add this to the loss for the center frame, *c* (Fig 2C, bottom right):

$$L_{T}=\sum_{i}\;\sum_{t=c-\epsilon }^{c+\epsilon }|I^{\left(t\right)}\left[\rho_{i}^{\left(t\right)}\right]-I^{\left(c\right)}\left[\rho_{i}^{\left(c\right)}\right]|$$

Note that since the loss term is applied for the center frame only, it does not affect the results for the other frames despite registering all frames in the patch together. Additionally, this component of the algorithm requires registration (or approximate registration) of nearby frames, making it more appropriate in low-motion conditions or after initial coarse registration is complete.

### User intervention

Our pipeline allows a user to dramatically improve tracking quality in various ways by providing further supervision. Providing additional fully annotated frames will improve registration targets to better match descriptors from similar frames. Strategically selecting a new reference frame can have dramatic impacts on frame sorting as well, creating opportunities to form tighter clusters of parent-child branches.

Furthermore, when multiple reference frames are present, covariance of keypoints in those frames helps better define an implicit global spatial structure by modulating stiffnesses of the spring connections between neighboring keypoints, *k*_*ij*_. Any additional reference frames can provide more accurate covariances, and thus a spatial model that is more accurately tailored for that particular dataset.

*Partially* annotated frames are not used to seed sorted frame branches nor used to sample reference descriptors. Still, all user annotations present in the frame are utilized to improve the tracking quality of the remaining keypoints in that frame (Fig 2D).

Firstly, prior to gradient descent, displacements between any annotations in the child frame and their corresponding coordinates in the parent frame are used to interpolate a flow field, Φ, which serves as a rough model of the global motion between the two frames [15, 26, 40]. The flow field provides an estimated displacement between the two frames for the remaining unannotated keypoints which can be applied to their coordinates in the parent frame to initialize those keypoints closer to their new positions in the child frame. This is particularly helpful for pairs of parent-child frames with large motion between them. While randomly annotating a small number of keypoints distributed across the frame is effective, the flow field can always be improved in both precision and accuracy by adding more annotations for the child frame (S3 Fig).

Secondly, the spatial regularization during the optimization process, $L_{N}$, also makes good use of any partial annotations. The annotations are fixed in place, but the spring connections to their neighbors remain a crucial component of the backwards gradient calculations and helps to “pull” the connected keypoints into place.

To streamline the process of providing user supervision, we offer a browser-based graphical user interface that provides an intuitive, simple environment to produce and save further annotations. Since our approach lacks a slow “training” phase, any new annotations can be applied to tracking a frame directly from the GUI. A macro available in the GUI executes a temporary state of the algorithm quickly and efficiently, allowing users to see the precipitated improvements immediately.

Additionally, the GUI provides an opportunity for users to provide supervision without creating new annotations. The user may upgrade individual results into annotations or entire frames into new reference frames by marking them as correctly tracked. These user-confirmed frames will be treated as a regular reference frame next time the algorithm is executed, benefiting from all the improvements to tracking quality discussed previously. These improvements to the rest of the results can be observed immediately by executing the algorithm from the GUI. A high-level summary of this procedure is provided as Algorithm 1 below.

**Algorithm 1** ZephIR optimization loop

**for** *c* ∈ *sorted*_*frame*_*list* **do**

 **if** $\{k|k\in i,\theta_{k}^{\left(c\right)}\in annotations\}\neq \emptyset$ **then**

                 ⊳ Partial annotations

$\Phi \leftarrow \text{Interp}(\theta_{k}^{\left(c\right)}-\theta_{k}^{\left(p\right)})$ ⊳ Interpolate flow field

$\theta_{i}^{\left(c\right)}\leftarrow \theta_{i}^{\left(p\right)}+\Phi \left[\theta_{i}^{\left(c\right)}\right]$ ⊳ Initialize at prediction

 **else**

$\theta_{i}^{\left(c\right)}\leftarrow \theta_{i}^{\left(p\right)}$ ⊳ Initialize at parent results

 **end if**

 **for** *n* ← 1, *n*_*epoch* **do**

$L\left(\theta_{i}^{\left(c\right)}\right)\leftarrow \lambda_{R}L_{R}+\lambda_{N}L_{N}+\lambda_{D}L_{D}+\lambda_{T}L_{T}$

  **Backwards** L ⊳ Backpropagate gradients

  **Update** $\theta_{i}^{\left(c\right)}$ ⊳ Gradient descent

 **end for**

$\rho_{i}^{\left(c\right)}\leftarrow \rho \left(\theta_{i}^{\left(c\right)}\right)$ ⊳ Get keypoint coordinates

 **Write** $\rho_{i}^{\left(c\right)}$ ⊳ Save coordinates

**end for**

## Results

### Neurons in crawling worms (*C. elegans*)

Optical methods based on fluorescence activity of calcium binding indicators has become a standard tool for observing neuronal activity in *C. elegans*. To do so, it is necessary to track fluorescent signals from individual neurons across every frame in a recording. This poses a significant challenge, particularly when the animal is allowed to freely crawl. The worm’s brain undergoes fast, dramatic, nonaffine deformations, exhibiting a large variety (forward and backward motion, omega turns, coils, pharyngeal pumping, etc.) and magnitude (up to ten microns relative to an internal reference frame) of movements as the animal behaves [20, 47–49].

Many solutions have been proposed to track fluorescent neurons in *C. elegans*. Two step (detect and link) approaches often suffer from the lack of reliable detection algorithms and require relatively low frame-to-frame motion in order to accurately link the detected neurons [6, 14, 23]. Similarly, deep learning approaches are limited by insufficient training data, often failing to generalize across different animals, even those within the same strain [11, 25]. While these approaches have provided important insight and progress, there remains substantial need for improvement in accuracy and efficiency when tracking many neurons in freely behaving worms.

**Fig 3:**
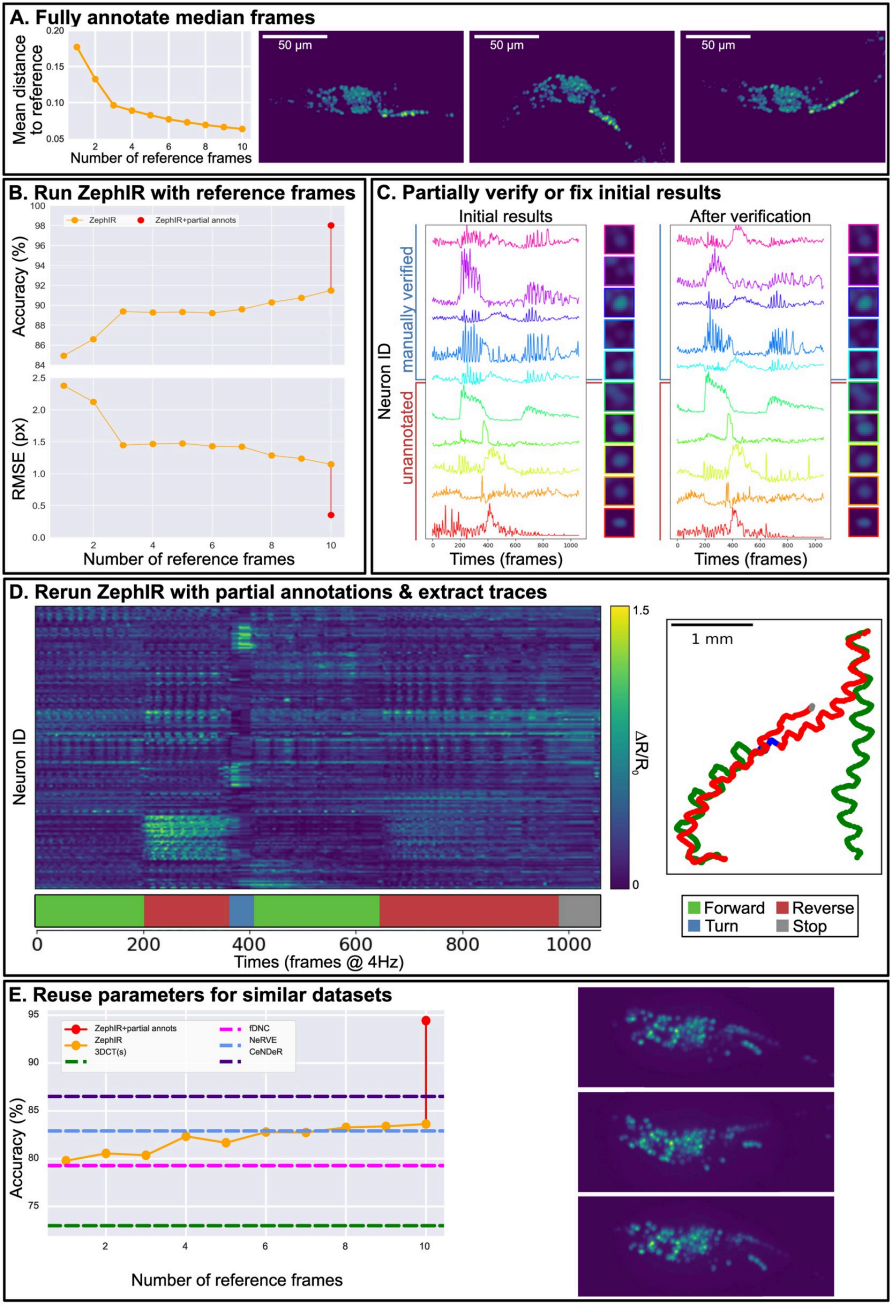
ZephIR analysis workflow and results for tracking GCaMP fluorescence from neuronal nuclei in 3D volumes of freely behaving *C. elegans*. **A:** Plot of mean distance (in similarity space) to the nearest reference frame vs the number of reference frames (left), and the first three median frames (maximum intensity projections of shape 200 x 512) recommended by ZephIR’s k-medoids clustering algorithm (right). The first three median frames clearly represent the three main postures that the worm cycles through as it crawls. **B:** Accuracy (higher is better) and precision (lower is better) vs the number of reference frames. Accuracy is measured as the average percentage of neurons correctly tracked, where a neuron is considered correctly tracked if the tracked coordinate is within the volume of the neuron as identified via a manual annotator. Precision is presented as the average RMS error between the predicted position and the manually annotated position of each neuron in pixels. Note that once the majority of the postures present in the data is well-represented by the first three reference frames, subsequent additions returns diminished improvements. Last data point shows ZephIR’s accuracy using 10 reference frames with 10 partial annotations across all frames (panel C). **C:** 10 neurons were randomly selected to be verified or corrected to serve as partial annotations. Traces of 5 of these neurons extracted using the initial ZephIR results with 10 reference frames (left), and those using verified true positions (right) are shown, along with 5 other randomly selected neurons. Traces are calculated as fold change of the ratio between GCaMP and RFP fluorescence of each neuronal nuclei over the baseline, where the baseline is defined as the ratio in the first frame. Tracking quality for these 10 neurons can also be seen in individual crops around the neurons averaged across all frames (sharper image of the cell at the center reflects better accuracy and precision in tracking). Note how the five unannotated neurons show improvements in tracking quality after the addition of partial annotations, exemplifying the effects of partial annotations on the unannotated neurons in the same frame. **D:** Neuronal activity traces from 178 neurons, extracted using results from ZephIR with 10 reference frames and 10 partial annotations in all frames. Traces are calculated as fold change of the ratio between GCaMP and RFP fluorescence of each neuronal nuclei over the baseline, where the baseline is defined as the ratio in the first frame. Behavior is shown in the ethogram below the heatmap. Trajectory of the worm (*t* = 0 at bottom right) is also colored with the behavior state at the time. Trajectory of the worm matches changes in behavior over time as expected, and many of the neuronal activity traces show strong correlation with behavior. **E:** Accuracy vs the number of reference frames for tracking 79 neurons in a publicly available dataset of freely moving *C. elegans* [14]. Since the spatiotemporal patterns in the data are similar to the previously tracked data, we can reuse the same parameters and follow the same procedure to track the 79 neurons in the head of the worm. Each volume has been centered and rotated but no further straightening has been done. Since this particular dataset has also been used to benchmark a number of recently developed algorithms, we may also compare ZephIR’s accuracy with Neuron Registration Vector Encoding (NeRVE) [14], fast Deep Neural Correspondence (fDNC) [11], 3DeeCellTracker [23], and CeNDeR [46].

Fig 3 describes ZephIR’s general analysis workflow and performance on tracking a set of 178 fluorescent neurons in the head of a freely behaving worm across a 3D recording of approximately 4.4 minutes (1060 frames @ 4Hz). We collected this data for the purpose of testing this algorithm using a microscope and technique similar to that described in [47]. The video has been centered and rigidly rotated to maintain a consistent orientation of the worm, but no further straightening has been done. With only a few manually annotated reference frames (78 mins/frame on average with the provided GUI), ZephIR already achieves state-of-the-art accuracy as reported in recently published works on neuron tracking [11, 14, 23, 46] (Fig 3B, Table 1), where accuracy is measured as the average percentage of neurons correctly tracked, and a neuron is considered correctly tracked if the tracked coordinate is within the volume of the neuron as identified via a manual annotator. First three median frames determined via k-medoids clustering and recommended for annotation as reference frames clearly represent the principal postures that are repeatedly sampled during locomotion (Fig 3A). Fully annotating those three frames (534 total annotations) is sufficient to produce tracking accuracy of 89.27%, comparable to previously published works on neuron tracking (Fig 3B, Table 1). Annotating additional median frames can increase this accuracy up to 91.49% for a total of 10 reference frames.

**Table 1:** Summary of results and performance of ZephIR and other recently developed algorithms for tracking neurons in freely moving *C. elegans*. When benchmarking on a common dataset (provided by [14]), ZephIR achieves top 2 accuracy with significantly less annotations and faster performance. Note that tracking inference speed does not include time spent for any potential training or detection.

Algorithm	Dataset source	Neurons labeled	Annotations used	Inference speed	Accuracy
ZephIR	This paper	178	3 reference frames	1.24 s/vol	89.27%
ZephIR	This paper	178	10 reference frames +10 partial	1.24 s/vol	98.02%
ZephIR	NeRVE [14]	79	10 reference frames +10 partial	1.08 s/vol	94.43%
CeNDeR [46]	CeNDeR C1 [46]	158	130 training frames	0.02 s/vol	95.48%
CeNDeR [46]	NeRVE [14]	69	130 training frames	0.01 s/vol	86.51%
NeRVE [14]	NeRVE [14]	69	100 frames	> 40 s/vol	82.9%
fDNC [11]	NeRVE [14]	69	2.304x10^5^ semi-synthetic training frames	0.01 s/vol	79.1%
3DeeCellTracker (single) [23]	NeRVE [14]	90	1 + 5x10^11^ synthetic training frames	73 s/vol	73%
3DeeCellTracker (ensemble) [23]	NeRVE [14]	90	1 + 5x10^11^ synthetic training frames	86 s/vol	99.8%

We further improve on the accuracy of the initial results by providing additional supervision. We randomly selected ten neurons uniformly distributed throughout the brain to verify and use as partial annotations across all frames (Fig 3C). Because the initial results already achieved high accuracy, they only required correction for a subset of frames (≈ 15%), taking an average of 51 mins/neuron of human time to complete across the entire movie. After this correction and validation, annotations for these ten neurons were re-classified as manual annotations in all frames. The partial annotations produce a dramatic improvement in accuracy (red data point in Fig 3B) without the need to verify entire frames.

Through this workflow, we are able to achieve a sufficiently high accuracy to extract neuronal activity traces across the entire recording (Fig 3D) [44, 45]. Neuronal activity is calculated as the average ratio of GCaMP and RFP fluorescence intensities of the 9 brightest pixels in a 3x7x7 volume (roughly the size of the cell nucleus in the image) centered around the tracked neuron coordinates after masking out pixels that overlap with the volumes around 5 nearest neighbors. Traces are adjusted for photobleaching (double exponential fit) and are shown relative to the ratio of intensities seen in the first frame. Many neurons show clear correlation with observed behaviors, and the activity patterns are comparable to previously published works [14, 49–51].

In order to verify and benchmark the accuracy of our method, we apply the same workflow to a similar dataset of a freely moving worm provided by Nguyen, *et al*. [14], a publicly available dataset with ground-truth results provided that has already been used as a benchmark for other recently published algorithms. [11, 14, 23, 46] Since this dataset is similar in behavior, motion, and imaging conditions, we may reuse the same parameters and follow the same workflow as the previously discussed *C. elegans* dataset. Doing so, we are again able to achieve state-of-the-art accuracy (84.0%) with only a few reference frames, and adding additional partial annotations increases our top accuracy further (94.48%) (Fig 3E). The top accuracy, the required annotations or training frames, and tracking inference speed (not including any training or detection) from other algorithms recently developed for tracking neurons in the same *C. elegans* dataset are summarized in Table 1.

### Comparison of loss terms and their combinations

**Fig 4:**
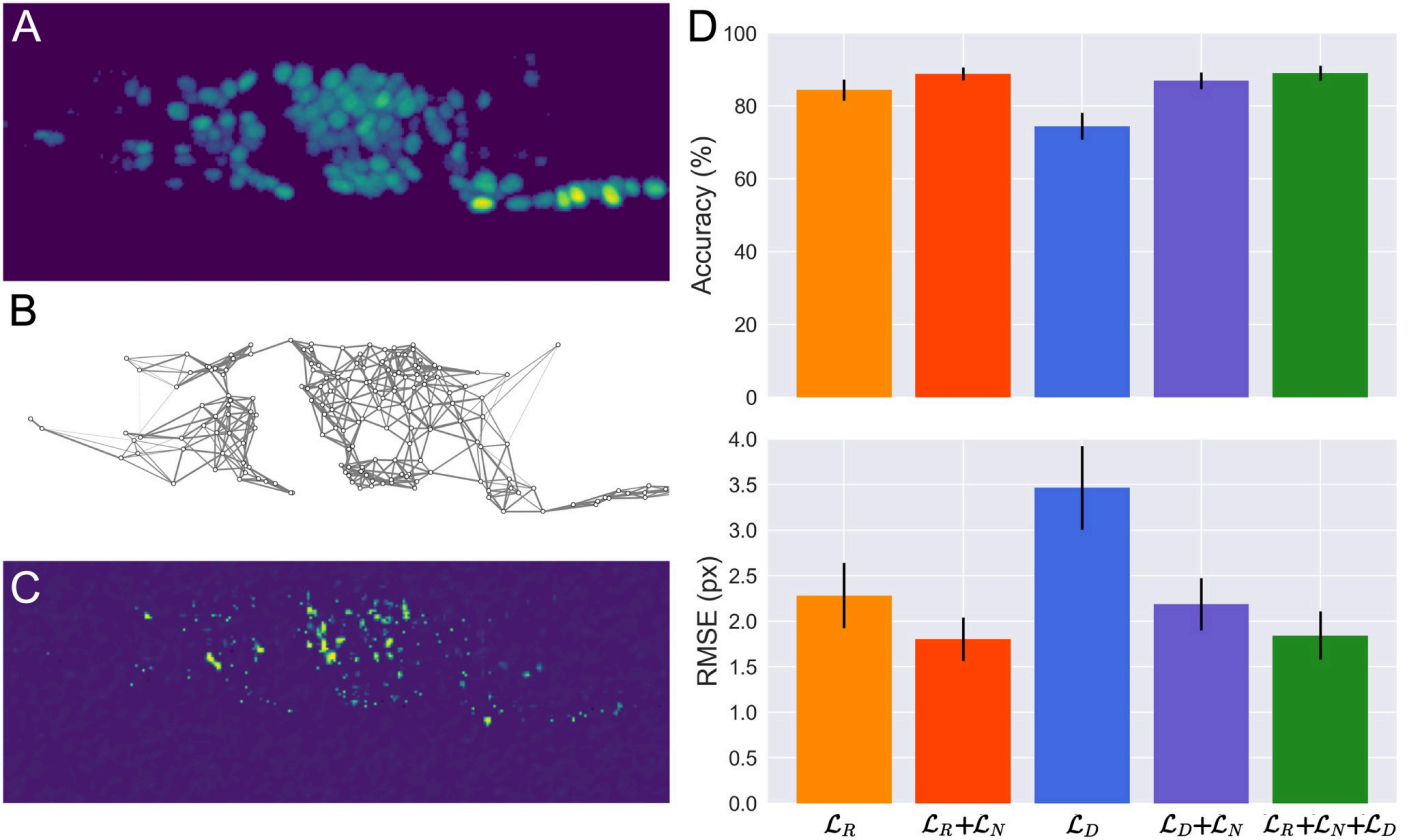
Results for tracking GCamP fluorescent neuron nuclei in 3D volumes of freely behaving *C. elegans* (same dataset as shown in Fig 3A–3D) with varying combinations for the tracking loss. Three reference frames and fixed weights (λ_*R*_, λ_*N*_, λ_*D*_) were used for all results shown. **A.** One of the three reference frames. **B.** Network of connections between neighboring neurons in the frame shown in panel A. The edge weights represent their relative stiffness for calculating the spring loss, $L_{N}$. The connections and their stiffness are modulated by the distance between the neurons and the covariance of the connected pair in reference frames. **C.** Results from feature detection, $L_{D}$, on the frame shown in panel A. Feature detection achieves an average precision and recall of 0.948 ± 0.024 and 0.931 ± 0.016, respectively. **D.** Tracking accuracy (top) and precision (bottom) as the loss components are changed. We can confirm the positive contributions of both $L_{N}$ and $L_{D}$ as adding either or both to image registration, $L_{R}$, increases accuracy and precision. We also note that $L_{D}$ have similar local information as the image descriptors when represented as a probability map with peaks at the neuron centers. When combined with the spring network, $L_{D}+L_{N}$, it reaches similar accuracy as image registration, and the sum of the three terms, $L_{R}+L_{N}+L_{D}$, result in only a small increase in accuracy compared to either combinations, $L_{R}+L_{N}$ or $L_{D}+L_{N}$.

By tracking the same set of keypoints in a single dataset with varying combinations for the tracking loss, we may examine how each component affects the accuracy and precision. We analyze the same dataset as discussed in the preceding section and Fig 3A–3D, and limit tracking to three reference frames with fixed weights and parameters (λ_*R*_, λ_*N*_, λ_*D*_). Fig 4 summarizes the resulting tracking accuracy and precision as the loss components are changed. We can confirm the positive contributions of both $L_{N}$ and $L_{D}$ as adding either or both to image registration, $L_{R}$, increases accuracy and precision (Fig 4B). We also note that $L_{D}$ have similar local information as the image descriptors when represented as a probability map with peaks at the neuron centers (Fig 4C). When combined with the spring network, $L_{D}+L_{N}$, it reaches similar accuracy as image registration, and the sum of the three terms, $L_{R}+L_{N}+L_{D}$, result in only a small increase in accuracy compared to either combinations, $L_{R}+L_{N}$ or $L_{D}+L_{N}$ (Fig 4D).

### Posture of a behaving mouse

Here, we demonstrate how ZephIR can be used for behavioral tracking in natural movies by analyzing the pose of a head-fixed mouse performing a motor task. The richness of local image features present in natural images lend themselves to registration. In addition, by connecting key points along the mouse’s body, our spring network loss ($L_{N}$) can implicitly capture the scaffold underlying the mouse’s posture.

There exist many solutions for similar problems in posture tracking. In particular, convolutional neural networks have been successfully implemented for posture analysis in both laboratory and natural settings [3–5]. Notably, DeepLabCut adapts a ResNet CNN architecture to track postures of various animals without any physical markers. DeepLabCut utilizes transfer learning, where a “base” model is trained on a publicly available dataset of various “natural” images prior to specializing the weights to a particular dataset. Their method reduces the amount of training data required to achieve state-of-the-art results by orders of magnitude (from hundreds of thousands to just a couple hundred labeled images), and thus reduces the amount of manual labor required by the experimentalist.

**Fig 5:**
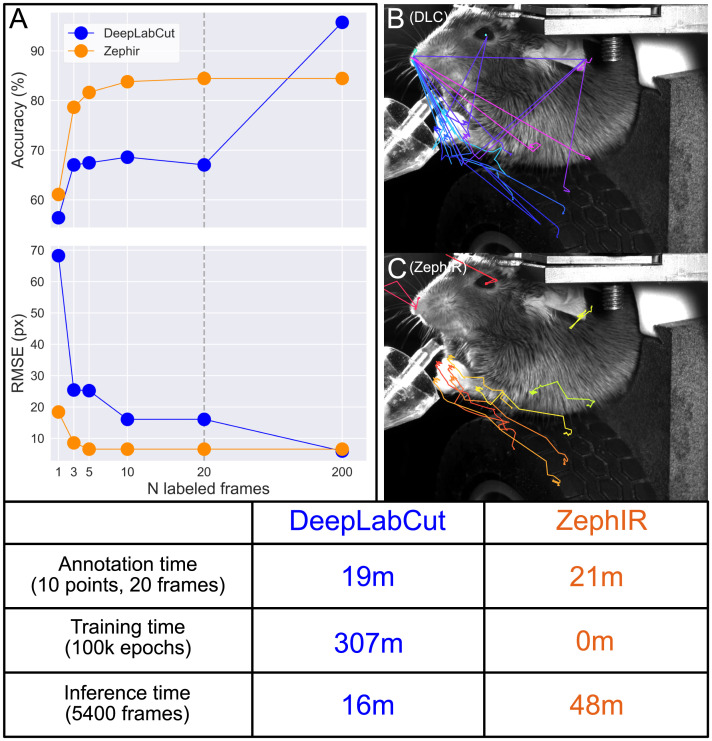
Results for tracking posture of a behaving mouse in 2D. We compare performances of ZephIR and DeepLabCut on tracking 10 body parts that characterize the mouse’s posture over time. **A:** Accuracy (average percentage of keypoints correctly tracked in unannotated frames) and precision (average distance between predicted position and ground truth position of keypoints) vs the number of manually labeled or ground truth frames. These labeled frames are used as reference frames for ZephIR and as training data for DeepLabCut. The frames are selected based on automated recommendations from each algorithm, meaning the two sets of frames used may not be identical. The last data point (results with 200 training frames) for DeepLabCut are produced with training data generated by verifying and correcting ZephIR results with 10 reference frames. Note that ZephIR achieves better accuracy when only a few labeled frames are provided, but DeepLabCut ultimately reaches a higher accuracy when its training data was augmented with ZephIR. **B, C:** DeepLabCut and ZephIR results with 20 labeled frames (vertical line in panel A) for tracking mouse body parts as it raises its paws. Note that ZephIR is more stable during motion while DeepLabCut tends to jump between the different body parts. **Table:** Annotation and computation speed comparison. Annotation time is calculated for the same person, using the respective GUI’s provided with each software package. Training and inference times are tested on the same CPU and single GPU environment and with 20 reference frames (vertical line in panel A). While DeepLabCut is faster for inference, it requires a slow training phase, dramatically increasing the total computation time. This data was produced and provided by the Churchland Lab (UCLA). Raw data is available at: https://ibl.flatironinstitute.org/public/churchlandlab/Subjects/CSHL047/2020-01-20/001/raw_video_data/.

Fig 5 compares the performance of our algorithm and that of DeepLabCut on the same dataset. This dataset of a mouse performing a motor task was produced by the Churchland Lab and published in [52]. We track ten points that summarize the mouse’s posture as it performs the task in a 2D movie with a total of 5400 frames. We show that for low numbers of reference frames, i.e. low numbers of training data, ZephIR can produce much better quality tracking than DeepLabCut, achieving good results with less than 20 reference frames (Fig 5B and 5C). ZephIR is also able to produce this result with significantly less total computation time as it does not require a slow training phase (table of Fig 5, middle row).

It is important to note that DeepLabCut can ultimately produce more accurate results when provided with more training data as seen when using 200 manually labeled frames (right-most data points in Fig 5A). While we do not expect ZephIR to replace DeepLabCut in such applications where a higher accuracy than what ZephIR is able to provide on its own is required, ZephIR can easily fit into a DeepLabCut workflow to augment the amount of training data available. Instead of manually labeling the full list of 200 frames to produce the last data point in Fig 5A, we only annotated the first 10 of the recommended frames. We then run ZephIR using those frames as references, verify the tracking results for the remaining 190 frames, and correct any errors to produce the full set of 200 training images to use for DeepLabCut. Including this step cuts the total human time required for a DeepLabCut workflow, from an extrapolated 160 minutes to label 200 frames by hand to 53 minutes.

More recently developed variants of DeepLabCut, such as DeepGraphPose, can also reduce training data size by incorporating spatio-temporal priors and enabling semi-supervised training that uses both annotated and unannotated data [5]. However, these variants still require a significant amount of training data ($\approx \frac{1}{3}$ of DeepLabCut’s requirements, compared to ZephIR’s ≈ 5 − 10%) and often fail when analyzing sparse or volumetric datasets, making it difficult to employ for biological datasets.

### Performance

We are able to compute the loss terms and optimize the tracking parameters efficiently by utilizing modern deep learning tools with, in particular, differentiable grid sampling and GPU acceleration offered by PyTorch [53]. Since our approach does not require a training phase, which is often the most significant bottleneck in both time and resources, it is fast without being computationally costly. We also sacrifice a small amount of performance to reduce the amount of memory required for both CPU and GPU to levels that are reasonable for commercial laptops. This balance can be manually adjusted by the user depending on their computing environment.

We run the following tests on a PC with a 16-core AMD Ryzen Threadripper 1950X processor @ 3.40GHz, 64GB RAM, and an Nvidia GTX 1080Ti GPU, 11GB VRAM. The tests were carried out on the freely behaving worm dataset (Fig 3).

In the default configuration, ZephIR registers 178x1x5x25x25 (*N*x*C*x*D*x*H*x*W*) descriptors ($L_{R}$) with spatial regularization ($L_{N}$) over 40 optimization epochs for an average of 1.24s total computation time spent per volume. In comparison, similar algorithms such as NeRVE takes an approximate 50 s/vol on over 200 computing cores [14] and 3DeeCellTracker approximately 1 min/vol on a desktop PC with an NVIDIA GeForce GTX 1080 GPU (inference only) [23].

During the test, the process utilized a maximum of 1.84GB RAM and 0.89GB VRAM. The number of descriptors does not significantly affect performance as descriptors are registered in parallel, but the number of epochs will impact speed linearly. The size of descriptors may slightly affect performance as well as memory consumption. The number of volumes in the dataset also affect the speed of frame sorting (Fig 1). When sorting frames by similarity (i.e. minimizing distance between parent and child frames), performance can be affected as *O*(*N*^2^), adding approximately 2.85 minutes to sort 1060 frames of shape 23 x 512 x 512.

## Availability and future directions

ZephIR is available at: https://github.com/venkatachalamlab/ZephIR.

The data acquired for Figs 3 and 4 that support the findings of this study are available at: (TBD prior to publication on zenodo.com).

ZephIR is a semi-supervised multiple object tracking algorithm. It tracks a fixed number of user-defined keypoints by minimizing a novel cost function that dynamically combines image registration, feature detection, and spatio-temporal constraints. Local registration of image features enables tracking of keypoints even in sparse imaging conditions, such as fluorescent cellular data, while a spring network incorporates a flexible motion model of the neighboring keypoints without the need for a highly specialized skeletal model. Feature detection can help fine-tune tracking results to match a nearby detected feature in the image or even recover good tracking accuracy in cases where registration clearly fails to produce good gradients. The model utilizes modern deep learning libraries, recent innovations in spatial transformers, and optimization tools to calculate loss and backpropagate gradients efficiently in a GPU environment.

We demonstrate that our approach is able to reach state-of-the-art accuracy on a diverse set of applications, including extracting neuronal activity traces in a freely moving *C. elegans* and tracking body parts of a behaving mouse. Notably, ZephIR is able to do so with a small amount of ground-truth data and low computational resource requirements. Recent deep learning-based methods often require large amounts of labeled frames for each new dataset. In contrast, ZephIR is able to generalize to radically different datasets with just a few labeled frames and adjustments to some hyperparameters.

Any amount of new manual labor, whether simply verifying correct results or fixing incorrect ones, can dramatically improve ZephIR’s accuracy. Verifying or correcting entire frames produces new reference frames to provide better reference descriptors for registration and improve flexibility of the spring network. Verifying only a subset of keypoints can initialize better tracking guesses for all other points in the same frame by interpolating a global motion model between parent and child frames. Additionally, any improvements in tracking a frame can cascade down to all its child frames, further reducing the amount of supervision required.

Through this workflow, ZephIR achieves state-of-the-art accuracy with minimal manual labor, even on a freely behaving *C. elegans*, where large deformations present a challenging tracking problem. We also expect to achieve similarly strong performance on sparse fluorescent videos of deforming neurons in other models organisms including *Hydra* (S6 Fig), zebrafish, and *Drosophila* [1, 54–56]. While its versatile design makes it useful for a diverse set of applications, given the emphasis on local image descriptors with a flexible spring network connecting neighboring keypoints to incorporate both global and local spatial information, ZephIR particularly excels when tracking high-contrast keypoints in sparse datasets with correlated and often repeating motion, such as fluorescent cells during locomotion. In addition, its semi-supervised workflow and ability to incorporate both partially and fully annotated frames allow the user to raise the tracking accuracy efficiently without the need for a slow training phase, making it a practical approach to tackle problems without a large corpus of data readily available.

With its versatile design and low computational requirements, ZephIR is designed to be highly accessible without requiring a dedicated workstation. On the other hand, we hope to also support full utilization of more powerful computational environments, especially when multiple GPUs are available. In particular, since distinct frame branches do not interact with one another when tracking, we may split them across multiple machines or GPUs to analyze in parallel, resulting in roughly linear gains in speed. These performance gains could be available to all users by hosting an updated version of our annotator GUI on a dedicated GPU server.

A notable limitation of our approach is that at least one annotated frame is required. We hope to mitigate this issue through future key upgrades. For example, an object detection algorithm may be able to automatically annotate the first reference frame, where linking or identity-classification is not necessary [7, 10, 13, 30, 57]. Many experiments with immobilized animals or low-motion data often only need one reference frame, meaning such datasets could be tracked entirely unsupervised. Advancements in spatial transformers and novel motion models may also eliminate or reduce the need for partial annotations to initialize keypoint coordinates closer to their true positions than the parent coordinates alone [2, 15, 24].

Similarly, improvements in feature detection may allow better automated determination of hyperparameters. Currently, the user will often need to adjust a number of parameters from the provided default values to improve tracking in a particular dataset, including the relative weights of the individual loss components whose typical values and range of values vary across datasets depending on the density of features, size of descriptors, etc. A feature detection and analysis model could instead supply the correct parameters prior to tracking, reducing the need for additional trial and error of determining the optimal set of parameters.

For some datasets, other approaches may be more accurate than ZephIR. As the field of deep learning continues to develop, we can expect more powerful, generalizable models to emerge. Still, ZephIR can be a powerful data augmentation tool upstream of any of these algorithms, as was demonstrated with behavioral mouse data in this work. Since it can reach reasonable accuracy with a low number of annotations, ZephIR can reduce the amount of labor required to produce the necessary training data. It may be a key component in generating a critical amount of ground-truth data to build new models to perform multi-object tracking in particularly challenging datasets.

## Supporting information

S1 FigVisualization of the loss map around certain neurons in freely moving *C. elegans* with all other neurons fixed at the ground truth position.Columns, from left to right: visualization of the volume around the neuron, the image descriptor of the neuron used for registration, map of the registration loss, map of the spring network loss, map of the feature detection loss, and map of the sum of the three previous losses. First column is centered at the initial coordinates, all others at the final optimized coordinates. Each row analyzes a different neuron and its optimization trajectory: initialized at red, optimized along orange, final results at green. Purple marks the ground truth position for the neuron. Comparing different loss maps along with the overall optimization trajectory can help diagnose certain tracking issues and give valuable insight on how to optimize the loss weights, **λ**, for a particular problem. **A:** For this neuron, all three loss components provide good minima at the ground truth position. ZephIR easily finds the correct result through gradient descent. **B:** For this neuron, ZephIR fails to escape a local minima present in both registration loss ($L_{R}$) and feature detection loss ($L_{D}$) at a neighboring neuron. However, we can see that spring network loss ($L_{N}$) creates good gradients that could push the neuron out of initial basin, thus increasing λ_*N*_ may improve this result. **C:** For this neuron, ZephIR fails to escape a local minima at a neighboring neuron. In contrast to row B, the spring network loss ($L_{N}$) contributes to this local minimum, but the registration loss ($L_{R}$) provides gradients towards a basin at the correct position. Thus, decreasing λ_*N*_ may improve this result. **D:**. For this neuron, all three loss components fails to present global minima at the ground truth position, and only the registration loss ($L_{R}$) presents a local minimum there. Since the neuron position is initialized such that it must cross a deeper minimum to reach the correct position in all loss maps, adjusting **λ** alone may not be able to improve this result.(EPS)

S2 Fig Verifying reference frame recommendation. ZephIR recommends frames to annotate as reference frames via k-medoids clustering of low-resolution thumbnails. We test ten candidate frames and all pairwise combinations. During clustering, each child frame is assigned to a cluster around a reference frame based on minimum distance (i.e., assigned to nearest reference frame). With each update, the clustering minimizes a score based on the mean distance between child frames and their assigned reference frame (left, lower/bluer is better). The results from tracking with the two candidate reference frames are evaluated for all other frames (right, lower/bluer is better). We can compare the resulting profiles of score and accuracy for each pair of candidate frames in order to evaluate the efficacy of the recommendation method (more similar is better).
(EPS)

S3 Fig Verifying parent-child selection. When sorting based on frame similarity, each subsequent child frame is selected to minimize the distance from a parent frame. We test pairs of frames to study the effect of distance between parent and child frames. In these tests, the parent frames provide both the initial positions of the keypoints in the child frame and the reference descriptors as registration targets, and tracking results for keypoints in the child frame are evaluated. It is evident in the resulting curve that accuracy quickly falls with distance.
(EPS)

S4 FigTesting sensitivity to weight (λ_*N*_) of spring network loss ($L_{N}$).We calculate the tracking accuracy (top) and precision (bottom) for tracking 178 neurons in freely moving *C. elegans* (see Fig 3) using 3 reference frames. The weight of the spring network loss is adjusted while all other hyperparameters remain fixed. Within a single order of magnitude, the effect of the hyperparameter on the accuracy and precision is small (<2%). However, it is important to note that continuing to increase the loss coefficient begins to deteriorate the accuracy, most likely due to exploding gradients during optimization. The vertical line indicates the default value, 1.0.(EPS)

S5 Fig Testing motion prediction. We evaluate tracking accuracy (top left, higher is better) and precision (bottom left, lower is better) for keypoints in a child frame (bottom right) as we add partial annotations. In these tests, we use another frame (top, middle right) as both the parent (providing initial positions for keypoints) and reference frame (providing reference descriptors for registration). We compare the improvements in performance from using displace vectors sampled from ZephIR’s interpolated flow field (top right) and those sampled from taCNN’s low-frequency deformation field (top middle).
(EPS)

S6 Fig Freely deforming Hydra. We track 50 neurons across 1000 frames with four reference frames. Each panel shows a trail of the neurons’ motions for the 100 frames preceding the frame shown.
(EPS)
